# Rocket-Like Microneedle Patch for Sustained Hormone Therapy in Prostate Cancer

**DOI:** 10.1021/acsbiomaterials.5c00127

**Published:** 2025-07-18

**Authors:** Ying-Tzu Chen, Kuan-Ta Chen, Kai-Jie Yu, See-Tong Pang, Hung-Wei Yang

**Affiliations:** 1 Department of Neurosurgery, Chang Gung Memorial Hospital, Linkou Branch, Taoyuan 33305, Taiwan; 2 Department of Biomedical Engineering, 34912 National Cheng Kung University, Tainan 70101, Taiwan; 3 Institute of Medical Science and Technology, 34874 National Sun Yat-sen University, Kaohsiung 80424, Taiwan; 4 Division of Urology, Department of Surgery, 38014 Chang Gung Memorial Hospital at Linkou Branch, Taoyuan 33305, Taiwan; 5 School of Medicine, College of Medicine, Chang Gung University, Taoyuan 33302, Taiwan; 6 Graduate Institute of Clinical Medical Science, College of Medicine, Chang Gung University, Taoyuan 33302, Taiwan; 7 Medical Device Innovation Center, 34912 National Cheng Kung University, No. 1, University Rd., Tainan City 70101, Taiwan

**Keywords:** microneedle patch (MNP), hormone therapy, sustained drug release, zeolitic imidazolate framework-8 (ZIF-8), prostate cancer (PCa)

## Abstract

Prostate cancer (PCa) remains a significant global health challenge, necessitating the development of advanced therapeutic strategies. This study introduces a rocket-like microneedle patch (RLMNP) as a dual-layer drug delivery system for sustained hormone therapy in PCa. RLMNP integrates a rapid-release layer, employing the pH-responsive zeolitic imidazolate framework-8 (ZIF-8) to encapsulate bicalutamide (CDX), and a sustained-release layer comprising an alginate-tyramine (Alg-Tyr) hydrogel loaded with goserelin. The dual mechanism synergistically targets PCa by blocking androgen receptor signaling and suppressing androgen synthesis. RLMNP demonstrates robust mechanical properties, efficient skin penetration, and controlled drug release profiles. *In vivo* studies highlight its remarkable efficacy in reducing tumor volume, extending survival (from 34 to 46 days), and mitigating side effects in PCa models. Furthermore, this painless, self-administered platform addresses key limitations of current hormone therapies, including low patient compliance and treatment-associated discomfort. By combining state-of-the-art microneedle technology and a dual-action therapeutic approach, RLMNP establishes a promising paradigm for PCa management. This innovation not only improves therapeutic outcomes but also sets the stage for broader applications of microneedle-based transdermal drug delivery in oncology.

## Graphical abstract

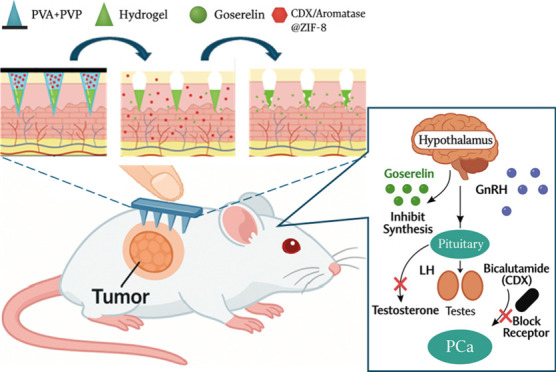

## 1 Introduction

According to the International Agency for Research on Cancer (IARC) statistics for 2022, prostate cancer (PCa) is the second most commonly diagnosed cancer and the fifth leading cause of cancer-related deaths among men worldwide, with 1.47 million new cases and 397,000 deaths reported. Driven by population growth and aging, the global burden is expected to rise significantly, reaching an estimated 2.9 million new cases and 827,000 deaths per year by 2045.^1^ Key risk factors include age, genetics, and family history, with age being the most critical—PCa is rare in men under 45 and becomes increasingly common with advancing age. Consequently, its prevalence is higher in developed countries with greater life expectancies.^2–4^ Treatment strategies are based on factors such as PSA levels, cancer stage, patient age, and potential quality-of-life impacts. Available options include active surveillance, surgery, chemotherapy, radiotherapy, cryoablation, focused ultrasound, and hormone therapy.^3,4^

Androgens, particularly testosterone and dihydrotestosterone (DHT), are key drivers of PCa progression. Androgen deprivation therapy (ADT)—through surgical or chemical castration—suppresses androgen signaling and remains a cornerstone of treatment.^5,6^ Chemical castration works by inhibiting androgen synthesis or blocking androgen receptor activity, but has drawbacks such as high cost, repeated clinical visits, and discomfort from injections.^7–10^ Oral agents face additional limitations due to first-pass metabolism, which reduces drug efficacy.^11,12^ These challenges underscore the need for a painless, user-friendly formulation paired with an innovative delivery system to enhance therapeutic outcomes and patient compliance.

Microneedle patches represent an innovative transdermal drug delivery system, consisting of microscopic needle structures integrated with a supporting backing layer. Since the stratum corneum—the outermost layer of the epidermis—is approximately 10 to 20 μm thick, microneedles are typically designed to exceed 15 μm in length to ensure effective penetration through this barrier. Unlike traditional hypodermic injections, microneedle application causes minimal pain, often described as a sensation akin to a gentle graze of Velcro on the skin. Furthermore, microneedle-based transdermal drug delivery systems circumvent the first-pass metabolism, thereby significantly enhancing drug delivery efficiency.^13,14^ The mechanical strength, stability, drug loading capacity, and release kinetics of microneedles are primarily determined by the properties of the base materials, including their solubility, molecular weight, viscosity, and concentration. The chosen materials must exhibit long-term controlled-release characteristics to maintain drug concentrations within the therapeutic window over an extended period and achieve sustained therapeutic effects. For example, microneedles composed primarily of polylactic acid (PLA) and poly­(lactic-*co*-glycolic acid) (PLGA) and loaded with the contraceptive hormone levonorgestrel (LNG) were shown to maintain plasma hormone concentrations above therapeutic levels in humans for at least one month.^15^ Additionally, hydrogel-based microneedles offer the advantage of tunable drug delivery efficiency, which can be adjusted by modifying the cross-linking degree.^16^ Hydrogels are widely applied in biomedical fields due to their elasticity, adhesiveness, biocompatibility, and biodegradability. Applications include drug delivery, biosensors, tissue engineering, wound dressings, and blood-compatible coatings.^17^ Among hydrogel materials, alginate (Alg) is one of the most commonly utilized for drug delivery in biomedical applications.

Alg, a natural polysaccharide, exhibits a range of beneficial properties, including antiallergic effects (e.g., inhibition of serum histamine release), immune-enhancing activity (through polysaccharide-mediated activation of immune receptors), antioxidant functions (such as free radical scavenging, metal chelation, and ferric ion reduction), and anti-inflammatory effects.^18^ In addition, Alg is highly hydrophilic, biocompatible, and biodegradable,^19^ making it an ideal material for diverse biomedical applications. In drug delivery, Alg hydrogels offer distinct advantages over other polymers, particularly in the encapsulation of peptide- and protein-based therapeutics under mild conditions, which helps preserve protein activity and avoid denaturation. Furthermore, Alg hydrogels eliminate the need for organic solvents during cross-linking and allow for tunable drug release kinetics by adjusting the degree of cross-linking—higher cross-linking leads to slower degradation and more sustained release.^20,21^ Leveraging these advantages, this study employs Alg to fabricate dissolvable microneedles as drug delivery platforms. Most dissolvable microneedles consist of a single-material matrix or incorporate composite nanocarriers, but dual-layer microneedle systems are an emerging design with promising therapeutic applications. For example, Seok Chan Park et al. developed a biphasic dual-layer microneedle system for the controlled delivery of proteins such as growth factors and hormones. This design features a water-soluble base layer for rapid release and a biodegradable needle tip for sustained drug delivery.^22^ Similarly, Qu et al. introduced a multifunctional dual-layer microneedle combining nanohydroxyapatite (nHA) and gelatin methacryloyl (GelMA) in the base with a Mg-based metal–organic framework (Mg-MOF) in the tip to promote periodontal tissue regeneration.^23^ Liu et al. further demonstrated a dual-layer microneedle capable of releasing nitric oxide (NO) and oxygen (O_2_) to enhance diabetic wound healing by modulating neurovascular coupling and immune responses.^24^ These examples highlight the growing interest in dual-layer microneedles for precise and programmable drug delivery.

In parallel, nanoparticles have attracted significant attention in cancer therapy due to their enhanced permeability and retention (EPR) effect and potential for targeted drug delivery.^25–27^ Recent research has emphasized stimulus-responsive nanoparticles, particularly those that respond to acidic environments commonly found in tumors and intracellular compartments.^28^ Among them, zeolitic imidazolate frameworks (ZIFs) have emerged as exceptional carriers due to their high surface area, pH-responsiveness, biocompatibility, simple and rapid synthesis, chemical stability, water dispersibility, tunable pore size, and modifiability for targeted delivery.^29–31^ ZIFs remain stable at physiological pH (7.4) but rapidly degrade under acidic conditions (pH < 6) through protonation of 2-methylimidazole (2-MIM), destabilizing the framework and triggering drug release.^32,33^ These materials can also protect enzyme activity, improve storage stability, enable reuse, and achieve high loading capacities, including for hydrophobic drugs—further supporting their potential in cancer drug delivery.^34^

This study aimed to develop a dual-layer microneedle delivery system capable of simultaneously delivering two drugs with distinct mechanisms of action, offering an innovative combination therapy for PCa. The proposed system, termed the rocket-like microneedle patch (RLMNP), was engineered with a sustained-release long-acting layer and a rapid-release short-acting layer. The long-acting layer comprised a biocompatible, slow-degrading hydrogel incorporating the clinically approved drug Goserelin. Goserelin acts on the pituitary gland to effectively block the upstream synthesis of male hormones, thereby inhibiting hormone-driven tumor proliferation.^35^ In contrast, the short-acting layer utilized a pH-responsive zeolitic imidazolate framework-8 (ZIF-8) to encapsulate Bicalutamide (CDX), an androgen receptor inhibitor. Bicalutamide competitively binds to androgen receptors on PCa cells, disrupting tumor growth signals and inducing apoptosis (Scheme 1).^36^ The short-acting layer, located near the microneedle base, was designed for rapid dissolution upon application, enabling immediate drug release and action. Meanwhile, the long-acting layer penetrated the stratum corneum and remained stable within the body, providing sustained drug release while minimizing residual drug on the microneedle patch. By incorporating drugs targeting different therapeutic pathways, the RLMNP achieved enhanced efficacy through its synergistic formulation. Additionally, the painless and user-friendly nature of the microneedles addressed challenges related to low patient adherence and the generation of medical waste, offering a novel and practical drug delivery platform for PCa patients requiring hormone therapy.

**Scheme 1:**
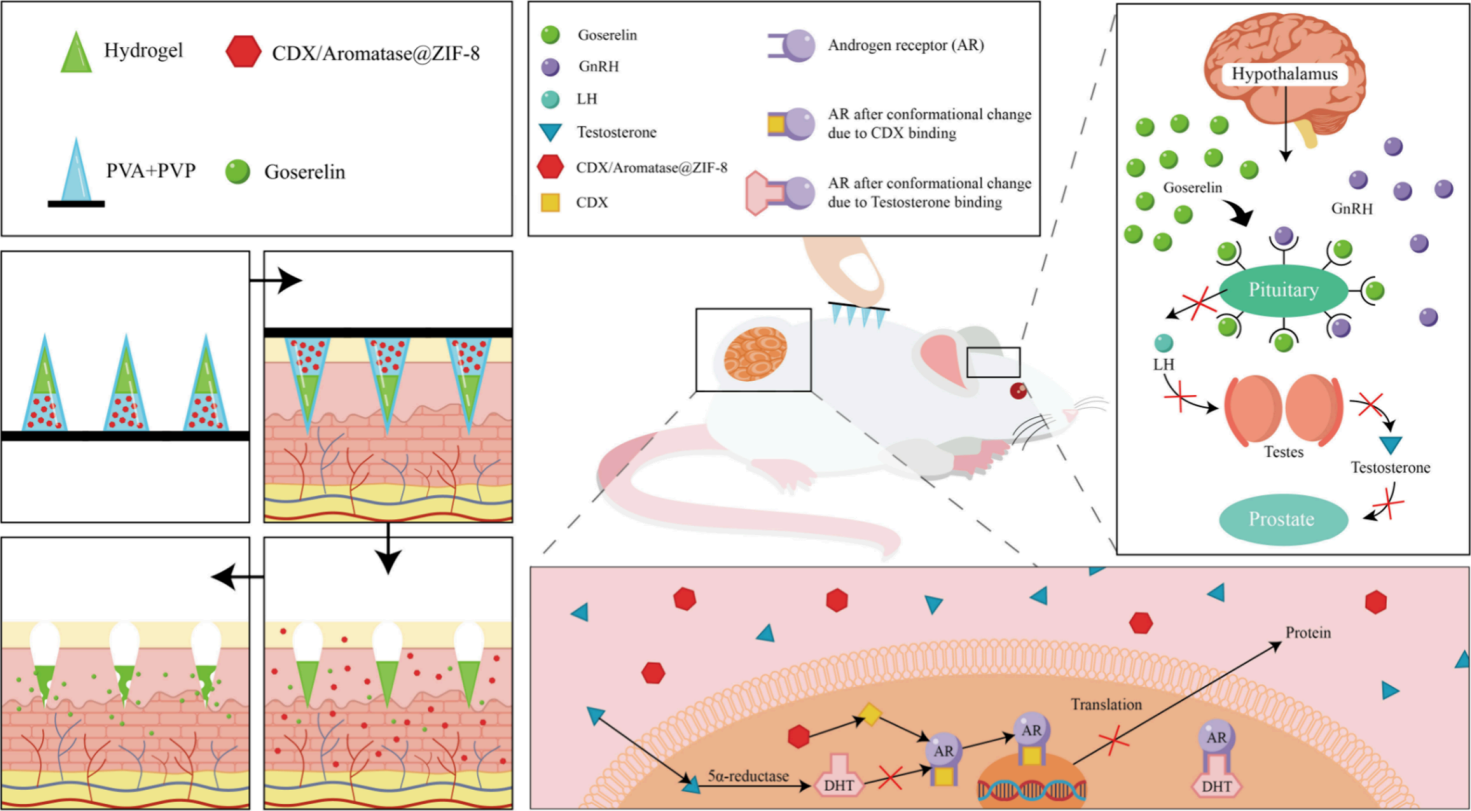
A Schematic Illustration Depicting the Hormonal Treatment Mechanism of RLMNP Applied in a Mouse Model

## 2 Materials and Methods

### 2.1 Materials

The following materials were utilized in this study: hydrochloric acid, sodium alginate, 2-MIM, CypExpress aromatase, poly­(vinyl alcohol) (PVA; Mw: 6000), polyvinylpyrrolidone (PVP; Mw: 10,000), rhodamine B (RhB), zinc chloride (ZnCl_2_), Tyramine (Tyr), and fluorescein-5-isothiocyanate (FITC), all of which were procured from Sigma-Aldrich (St. Louis, MO, USA). Ethanol (∼99%) and 2-morpholinoethanesulfonic acid (MES) monohydrate (free acid, crystalline) were obtained from J.T. Baker (Pennsylvania, USA). *N*-hydroxysuccinimide (NHS) was sourced from Alfa Aesar (Ward Hill, MA, USA). Polydimethylsiloxane (PDMS), composed of Component A (base agent) and Component B (curing agent), was obtained from Sil-More Industrial Ltd. (New Taipei City, Taiwan).

### 2.2 Preparation of Hydrogel Monomer Structure

A total of 100 mg of Alg was dissolved in 7 mL of MES buffer (pH 5.5) under continuous stirring while heating at 55 °C until completely dissolved. Separately, 250 mg of Tyr was dissolved in 2 mL of MES buffer under stirring at 30 °C until fully dissolved. After both solutions cooled to room temperature, they were thoroughly combined. Subsequently, a solution containing 250 mg of EDC and 250 mg of NHS dissolved in 1 mL of MES buffer was added to the mixture. The reaction was conducted under light-protected conditions for 3 h. The resulting reaction mixture was gradually dropped into 100 mL of 95% ethanol. The precipitate was collected by centrifugation at 5,000 rpm for 3 min, followed by a reduction in centrifugation speed to 2,000 rpm to remove unreacted materials. The product was washed three times with ethanol and dried overnight in a vacuum oven. After complete drying, the dried Alg-Tyr hydrogel monomer was stored at 4 °C in a refrigerator.

### 2.3 Image Analysis of Sustained-Release Hydrogel before and after Cross-Linking

A fixed concentration of Alg-Tyr (10 wt %), Goserelin (6 μL, 1 mg/mL), and horseradish peroxidase (HRP) (6 μL, 2 mg/mL) was used, while the concentration of hydrogen peroxide (H_2_O_2_) was varied (5, 10, and 15 wt %). The prepared hydrogel mixtures were dispensed into the caps of microcentrifuge tubes and allowed to rest undisturbed for 10 min. Gelation was evaluated visually by testing the resistance to stirring with a pipet tip. To further confirm successful cross-linking, 200 μL of deionized water (ddH_2_O) was added to the hydrogels to assess the stability of the formed gel. To validate the modification of the hydrogel monomer, samples containing HRP and H_2_O_2_ (cross-linking) and those without H_2_O_2_ (non-cross-linking) were placed in glass vials and photographed under various orientations, including upright, horizontal, angled, and inverted positions.

### 2.4 *In Vitro* Release of Sustained-Release Alg-Tyr Hydrogel

The RhB (0.1 mg/mL) was employed as a model drug to replace Goserelin, and hydrogels were prepared within 12-well Transwell inserts. Prior to initiating the experiment, 1 mL of PBS (pH 7.4) containing 0.05% collagenase was added beneath each Transwell insert and left for 1 min to remove any unencapsulated RhB from the hydrogel surface. Subsequently, 1 mL of PBS containing 0.05% collagenase was added as the initial release medium. The plate was then placed in a 37 °C incubator and agitated at 200 rpm. Sampling was performed at the following intervals: every hour during the first 1–8 h and every 8 h from 24 to 120 h. At each sampling point, 200 μL of the release medium was collected and analyzed using a fluorescence spectrometer with an excitation wavelength of 546 nm and an emission wavelength of 568 nm. Each measurement was performed in triplicate, and all experiments were conducted in triplicate for each group (N = 3).

### 2.5 Preparation of ZIF-8 Nanoparticles

Two milliliters of 2-MIM (12 mg/mL), 500 μL of ddH_2_O, 500 μL of 95% ethanol, and 100 μL of 1% PVA were added to a sample vial and stirred for 10 min to ensure uniform mixing. Subsequently, 1 mL of ZnCl_2_ solution (5 mg/mL) was added dropwise under continuous stirring at 500 rpm. The reaction mixture was allowed to react for 6 h, then centrifuged at 13,000 rpm for 15 min. The speed was then reduced to ″slow″ to remove any unreacted materials. The resultant product was washed thrice using a 4:1 water and ethanol volume ratio. Finally, the washed product was dried overnight in a vacuum oven, yielding pure PVA@ZIF-8 in a dry state, which was stored at 4 °C in a refrigerator. The procedure was modified to synthesize CDX-encapsulated ZIF-8 (CDX/Aromatase@ZIF-8) by replacing 500 μL of ddH_2_O with 500 μL of aromatase (0.25 mg/mL) and 500 μL of 95% ethanol with 500 μL of CDX (1 mg/mL) during the initial mixing step. For the preparation of FITC/Aromatase@ZIF-8, 2 mL of 2-MIM (12 mg/mL), 500 μL of aromatase (0.25 mg/mL), 500 μL of FITC (0.03125 mg/mL), and 100 μL of 1% PVA were added to a sample vial and stirred for 10 min to ensure thorough mixing. Subsequently, 1 mL of ZnCl_2_ solution (5 mg/mL) was added dropwise under stirring at 500 rpm, and the reaction was allowed to proceed for 6 h. Upon completion, the product was isolated as FITC/Aromatase@ZIF-8.

### 2.6 Drug Release Study of ZIF-8

Approximately 1 mg of FITC/Aromatase@ZIF-8 was placed into microcentrifuge tubes containing 1 mL of PBS (pH 5.5 or 7.4). The samples were incubated in a dry bath shaker set to 37 °C and 200 rpm to facilitate the release of the encapsulated content. Due to the rapid-release design of FITC/Aromatase@ZIF-8, the release profile was monitored over 3 days, with sampling intervals of every hour during the first 8 h and every 12 h from 24 to 72 h. At each time point, 200 μL of supernatant was collected and transferred to a black 96-well plate. Fluorescence measurements were performed using a SpectraMax M2 microplate reader (Molecular Devices, USA) with an excitation wavelength of 492 nm and an emission wavelength of 518 nm. Before measurement, the plate was shaken for 5 s, and each sample was analyzed in triplicate. To determine the FITC content of FITC/Aromatase@ZIF-8, approximately 1 mg of the FITC/Aromatase@ZIF-8 was dissolved in 500 μL of ddH_2_O, followed by the addition of 500 μL of 1 M HCl. Measurements were conducted three times, and the average value was calculated.

### 2.7 Effectiveness Analysis of CDX/Aromatase@ZIF-8

In this study, the androgen-sensitive human PCa cell line LNCaP was utilized, along with the CWR22R cell line, which was cultured for animal experiments. Both cell lines were maintained in RPMI 1640 medium supplemented with 10% hormone-free fetal bovine serum (FBS) and 1% penicillin-streptomycin. The cells were incubated at 37 °C in a humidified atmosphere containing 5% CO_2_ for 24 h to prepare them for subsequent experiments.

To examine the concentration-dependent effects of the DHT on LNCaP and CWR22R cells, 8 × 10^3^ cells were seeded in 100 μL of medium per well in a 96-well plate and cultured for 24 h. Subsequently, 100 μL of an RPMI 1640 medium containing DHT was added to each well, with final DHT concentrations adjusted to 0.01 nM, 0.1 nM, 10 nM, and 100 nM. After a 48-h incubation, the medium was replaced with 100 μL of CCK-8 working solution (RPMI 1640: CCK-8 = 9:1). The cells were then incubated for an additional hour, and the absorbance at 450 nm was measured using a SpectraMax M2 microplate reader (Molecular Devices, USA) to assess cell viability. For further analysis, LNCaP and CWR22R cells were seeded in 96-well plates and cultured for 24 h following the same protocol as the cytotoxicity assay. The cells were divided into four groups: (1) DHT, (2) DHT + ZIF-8 (50 μg/mL), (3) DHT + CDX (10 μM), and (4) DHT + CDX/Aromatase@ZIF-8 (containing 10 μM CDX and 50 μg/mL ZIF-8). All treatment groups were supplemented with 100 nM DHT. After 24 h of coculture, cell viability was evaluated using the CCK-8 assay.

For biocompatibility evaluation, LNCaP and CWR22R cells (8 × 10^3^ cells per 100 μL) were seeded into 96-well plates and incubated at 37 °C with 5% CO_2_ for 24 h. The culture medium was then replaced with RPMI 1640 medium supplemented with Pure ZIF-8 at concentrations of 0, 10, 25, 50, 75, 100, and 125 μg/mL, followed by an additional 24 h of incubation under identical conditions. Subsequently, the medium was replaced with 100 μL of a CCK-8 working solution (RPMI 1640: CCK-8 = 9:1), and the cells were incubated for 1 h. Absorbance at 450 nm was measured using a microplate reader to assess cell viability relative to the control group. The results demonstrated the biocompatibility of the tested material.

### 2.8 Preparation of RLMNP

First, a high-resolution 3D printer (microArch S140, 10 μm Series) is employed to fabricate a microneedle master mold using HTL resin, which serves as the template for preparing PDMS molds. The 3D-printed microneedle array comprised a 10 × 10 configuration, with each microneedle exhibiting a conical shape, a tip diameter of approximately 5–10 μm, a base diameter of 500 μm, and an overall height of 840 μm. A total of 15 g of PDMS base was thoroughly mixed with 1.5 g of curing agent until a uniform and homogeneous mixture was obtained. The mixture was then degassed under vacuum for 30 min to remove entrapped air bubbles. Following degassing, the mixture was poured into a plastic container containing four 3D-printed microneedle master molds, ensuring complete coverage of each mold. The container was subsequently placed on a heating plate preheated to 70 °C and allowed to cure for 2 h. After complete curing, the PDMS was carefully demolded, and any excess material around the edges was trimmed to yield the final PDMS microneedle molds.

To fabricate single-layer microneedles, 1 g of PVA powder and 3 g of PVP powder were added to an empty sample vial, followed by 4 mL of ddH_2_O. The mixture was heated at 70 °C and stirred at 100 rpm overnight until completely dissolved, forming the PVA–PVP solution for the microneedle base. A coating solution was formulated to prepare needle tips by combining equal volumes of a 10% PVA solution and a 1 mg/mL RhB solution. The fabrication began by applying 6 μL of the PVA+RhB mixture onto a PDMS mold. The solution was evenly distributed and centrifuged at 4,000 rpm for 30 s, a process repeated twice. The mold was subsequently dried on a vacuum plate for 1 h. Next, 10 μL of the PVA–PVP mixture was applied to the mold and centrifuged at 4,000 rpm for 1 min, with the excess solution removed. This step was repeated with an additional 10 μL of the PVA–PVP mixture, which was centrifuged for 3 min. A final thin layer of 6 μL PVA–PVP solution was then applied and left to dry overnight on a vacuum plate. The following day, the microneedles were carefully demolded using an acrylic plate and further dried in a vacuum oven for 8 h to ensure proper hardening.

To fabricate single-layer microneedles with FITC/Alg-Tyr hydrogel tips, 6 μL of HRP (2 mg/mL) was evenly applied to the PDMS mold and centrifuged at 4,000 rpm for 1 min. This step was repeated once. Subsequently, 3 μL of a mixture containing Alg-Tyr, H_2_O_2_, and FITC was applied evenly onto the mold and centrifuged at 4,000 rpm for 10 min. The procedure was repeated, increasing the centrifugation time to 15 min. The mold was then dried on a vacuum plate for 2 h, completing the preparation of the tip layer. The base layer was fabricated using the same procedure as described above. After demolding, the microneedles were dried in a vacuum oven until fully hardened. This process yielded single-layer microneedles with FITC/Alg-Tyr hydrogel.

To fabricate RLMNP, 6 μL of HRP (2 mg/mL) was evenly applied to a PDMS mold and centrifuged at 4,000 rpm for 1 min. This step was repeated to ensure uniform distribution. Subsequently, 3 μL of a mixture containing Alg-Tyr, H_2_O_2_, and Goserelin was evenly applied to the mold and centrifuged at 4,000 rpm for 10 min. The process was repeated, with the centrifugation time increased to 15 min. The mold was then dried on a vacuum plate for 2 h, completing the preparation of the tip layer. Next, 3 μL of CDX/Aromatase@ZIF-8 was evenly applied to the mold and centrifuged at 4,000 rpm for 1 min. This step was repeated to enhance consistency. The mold was subsequently dried on a vacuum plate for 1 h. Following this, 10 μL of a PVA–PVP mixture was applied to the mold and centrifuged at 4,000 rpm for 1 min, with any excess solution carefully removed. The application of the PVA–PVP mixture was repeated using an additional 10 μL, which was centrifuged for 3 min. A final thin layer of 6 μL of PVA–PVP solution was applied and allowed to dry overnight on a vacuum plate. The next day, the microneedles were gently demolded using an acrylic plate and subjected to further drying in a vacuum oven for 8 h. This process yielded RLMNP loaded with Goserelin and CDX/Aromatase@ZIF-8.

### 2.9 Characterization of RLMNP

The fabricated RLMNPs were dried in a vacuum oven for approximately 8 h. Following this, a scalpel was carefully used to scrape the microneedles, exposing the cross sections of the Alg-Tyr hydrogel layer and the CDX/Aromatase@ZIF-8 layer. The cross sections were then analyzed using SEM.

For mechanical strength testing, the RLMNPs were attached to the movable metal disk of a universal testing machine using double-sided tape, ensuring that the needle tips were aligned downward toward the base. The testing parameters were set to apply a vertical force of 10 N, with a downward movement speed of 0.1 mm/min and a deformation recording rate of 0.01 mm/second. The microneedles gradually deformed as the vertical force was applied until they fractured at their maximum load-bearing capacity. The force required for fracture was recorded to evaluate the mechanical strength of the microneedles.

For penetration testing, the subcutaneous fat of pig skin was removed using a scalpel and trimmed to a size slightly larger than the microneedle array. The surface of the pig skin was cleaned with alcohol and allowed to dry for 3 min to ensure complete evaporation of the alcohol. The prepared pig skin was laid flat, and the microneedles were vertically inserted into the skin for 1 min before being withdrawn. The surface of the pig skin was then wiped with alcohol-soaked tissue to remove any residual dye. After cleaning, the pig skin was observed under an upright fluorescence microscope using both visible light and fluorescence imaging to visualize the puncture holes.

To evaluate the dissolution ability of RLMNPs, RLMNPs loaded with FITC/Alg-Tyr hydrogel and RhB/Aromatase@ZIF-8 were placed in microcentrifuge tubes containing 1 mL of either PBS or 0.05% collagenase solution. In the PBS group, the samples were left to stand for 3 min to allow the dissolution of PVA and PVP. In the collagenase group, the samples were incubated at 37 °C and agitated at 200 rpm in a dry bath shaker for 24 h. After incubation, both groups were centrifuged at 13,000 rpm for 1 min to remove the supernatant, followed by washing with 1 mL of PBS. This washing was repeated five times to remove residual FITC from the hydrogel surface and supernatant, leaving only FITC encapsulated within the hydrogel. Finally, 200 μL of PBS was used to resuspend the FITC/Alg-Tyr hydrogel needle tips, which were then mounted on a glass slide, covered with a coverslip, and blotted to remove excess PBS. The FITC/Alg-Tyr hydrogel needle tip morphology was observed under an upright microscope. The release behavior of RLMNPs was also monitored following subcutaneous penetration into agarose gel. Images were captured using an upright fluorescence microscope at specific time intervals: 0, 5, 10, 30, 60, and 180 min postinsertion.

### 2.10 Studies of *In Vivo* PCa Hormone Therapy Using RLMNP

The Institutional Animal Care and Use Committee (IACUC) at Chang Gung Memorial Hospital reviewed and approved the protocol for this study under approval number 2017031306. To evaluate the efficacy of the RLMNP developed in this study, three experimental groups were established: a control group, an empty RLMNP treatment group, and an RLMNP treatment group. Tumor growth and survival rate were assessed in the same cohort of animals (control: N = 7; empty RLMNP: N = 5; RLMNP: N = 5). Tumor volume was measured every 2–4 days, and survival was defined as the time until tumor volume exceeded 2 cm^3^ or spontaneous death. NOD/SCID mice were subcutaneously implanted with PCa cells (CWR22R) at a concentration of 10^6^ cells per mouse, with treatment initiated on day 7 postimplantation. Prior to each treatment session, the hair over the treatment area was removed using a razor, followed by a depilatory cream. The empty RLMNP or drug-loaded RLMNP was applied to the skin near the tumor site by exerting approximately 1.5 N of force on the back of the RLMNP using the thumb. The RLMNP was then secured in place with adhesive tape for 10 min before removal. Treatments were administered every 4 days (on Days 7, 11, 15, 19, and 23), totaling five sessions over the course of one month. The study end point was defined as the tumor size exceeding 2 cm^3^, at which point the mice were euthanized in accordance with ethical guidelines. Survival rate was defined as the proportion of animals that remained alive without meeting the predefined humane end point criteria at each observation time point. The implanted PCa cells were genetically engineered to express luciferase via a lentiviral vector. Following intraperitoneal injection of the substrate luciferin, the luciferase enzyme catalyzed a reaction that emitted bioluminescent light. This bioluminescence was detected at the tumor site using the *in vivo* imaging system (IVIS, Xenogen IVIS 200). The intensity of the bioluminescent signal corresponded to tumor size and cellular activity, with larger tumors or more active cell proliferation producing stronger signals. In each group, IVIS imaging was performed on three representative mice, serving as a qualitative assessment of tumor progression. Throughout the experiment, survival, tumor size, and cellular activity were evaluated using bioluminescence imaging and caliper measurements. Additionally, daily body weight changes were monitored to assess the potential toxicity of the materials.

### 2.11 Statistical Analysis

The data were expressed as the mean ± SD based on at least three independent experiments. Statistical analysis was performed using Student’s *t* test. Differences were considered statistically significant if *p ≤ 0.05.

## 3 Results and Discussion

### 3-1 Characterization of Alg-Tyr Hydrogel

In this study, RLMNP was fabricated using an Alg-Tyr precursor, which was synthesized by chemically conjugating Tyr to the Alg backbone. The subsequent addition of HRPand hydrogen peroxide (H_2_O_2_) facilitated cross-linking, forming an Alg-Tyr hydrogel, as illustrated in Figure 1A. The Fourier transform infrared (FT-IR) spectra confirmed the presence of aromatic rings from Tyr in the Alg-Tyr conjugate, as indicated by the characteristic C–C (in-ring) stretching vibration peak at 1507 cm^–1^, which was observed exclusively in the spectrum of Alg-Tyr. Additionally, a significant reduction in the intensity of the C = O stretching vibration peak at 1737 cm^–1^, corresponding to the Alg backbone, was observed after Tyr conjugation. These results provide clear evidence of the successful synthesis of the Alg-Tyr precursor (Figure 1B). Subsequently, two experiments were performed to evaluate the feasibility of Alg-Tyr hydrogel formation under different conditions. An 8 wt % Alg-Tyr solution was initially prepared in vials, followed by the addition of an HRP solution (2 mg/mL) either with or without 5 wt % H_2_O_2_. In the presence of H_2_O_2_, hydrogel formation was observed, with the Alg-Tyr hydrogel aggregating at the bottom of the vial. Conversely, no gelation occurred in the absence of H_2_O_2_, and the Alg-Tyr solution remained in a fluid state, freely flowing within the vial (Figure 1C). These findings confirm that free radicals generated by the HRP/H_2_O_2_ system are crucial for initiating the cross-linking of Alg-Tyr precursors, which is necessary for successful hydrogel formation.

**Figure 1:**
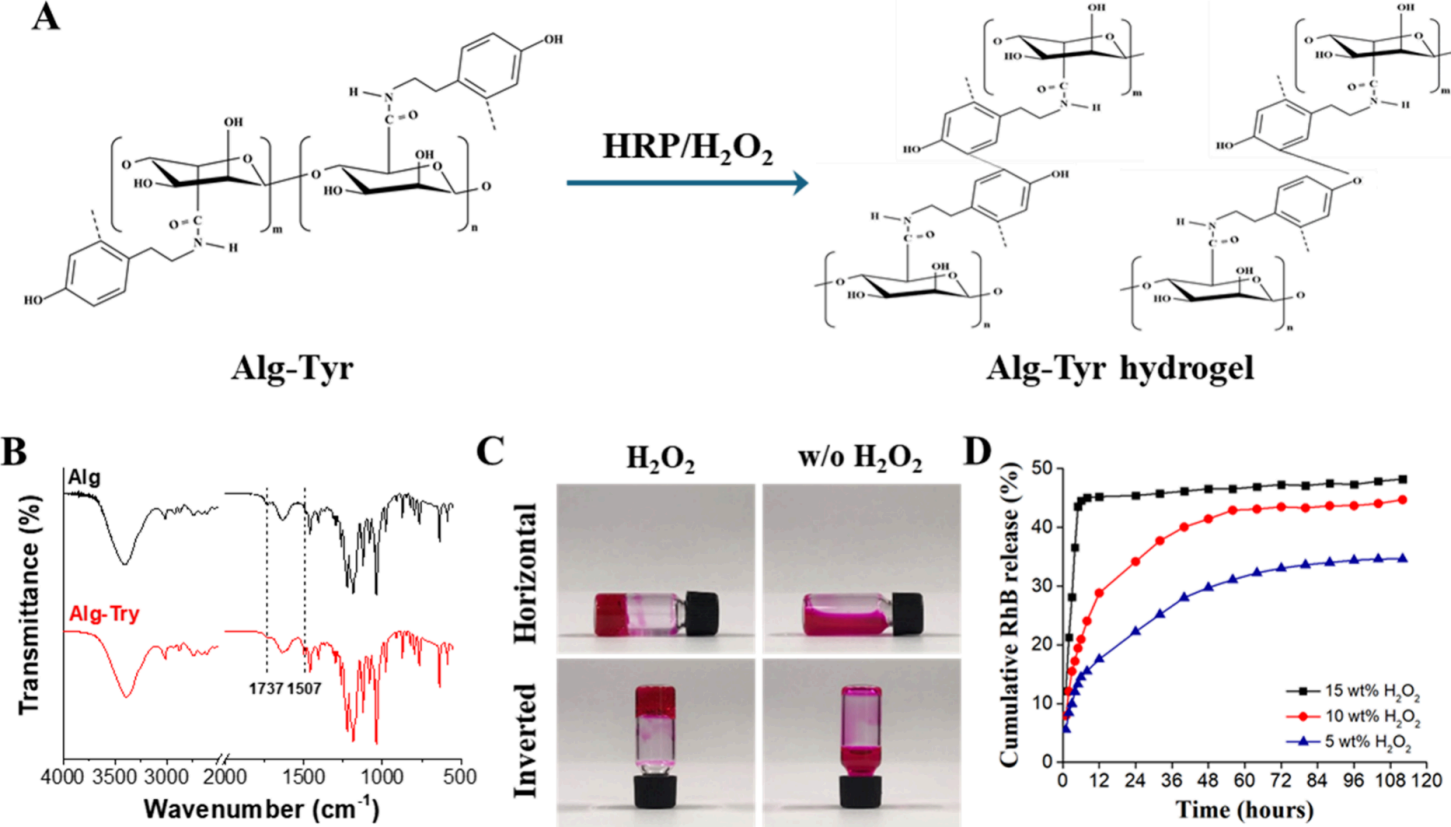
(A) Schematic representation of the synthesis of Alg-Tyr conjugates via carbodiimide coupling chemistry. (B) The FTIR spectrum of the Alg-Tyr conjugates confirms successful functionalization. (C) Photographs of the Alg-Tyr solution illustrate its gelation stability in the presence and absence of H_2_O_2_. (D) *In vitro* cumulative release profiles of Alg-Tyr hydrogels prepared with varying concentrations of H_2_O_2_.

The release of encapsulated drugs from the hydrogel network primarily occurs through two mechanisms: (1) diffusion of the drug into the surrounding environment via pores formed as the hydrogel swells; (2) release of the drug as the hydrogel degrades over time. Thus, in this study, we examined the influence of H_2_O_2_ concentrations (5, 10, and 15 wt %) on the cross-linking level of Alg-Tyr chains and its subsequent impact on drug release efficiency. The cumulative drug release profiles are depicted in Figure 1D. The hydrogel cross-linked with 15 wt % H_2_O_2_ exhibited an initial burst release, reaching approximately 44.5% within the first 6 h, followed by a total cumulative release of 48.2% after 112 h. This rapid release can be attributed to the excessive concentration of H_2_O_2_, which generates an overabundance of free radicals that inactivate HRP, thereby impairing the cross-linking process.^37,38^ In contrast, hydrogels prepared with 10 and 5 wt % H_2_O_2_ exhibited more sustained release profiles, with 42.9% and 34.1% cumulative release over 56 and 88 h, respectively, and final cumulative release efficiencies of 44.6% and 34.7% after 112 h. While the hydrogel prepared with 5 wt % H_2_O_2_ provided the longest sustained release duration of up to 88 h, its overall cumulative release remained relatively low. Collectively, the hydrogel prepared with 10 wt % H_2_O_2_ demonstrated the most balanced and favorable release characteristics. Therefore, 10 wt % H_2_O_2_ was selected as the optimal cross-linking concentration for hydrogel preparation in the subsequent fabrication of RLMNP.

### 3-2 Characterization of Zeolitic Imidazolate Framework-8 (ZIF-8)-Based Drug Carrier

ZIF-8 holds significant potential as a key material for drug delivery, primarily due to its exceptional capacity to encapsulate diverse small molecules and enzymes, effectively enhancing their structural stability. To preserve the activity of the drug or enzyme, the preparation of ZIF-8 must be carried out under mild conditions, specifically at room temperature, within a short time frame, and in an aqueous solution.^39^ However, in the absence of nucleating agents, forming well-defined and mature crystals under such stringent conditions is challenging, resulting in poor control over particle size and uniformity. Therefore, in this study, we investigate whether Aromatase can simultaneously function as both a drug and a nucleating agent. PVA is a biocompatible polymer that forms ordered chain bundles through thermal motion in solution, serving as nucleation sites for synthesizing ZIF-8 (PVA@ZIF-8). This is primarily attributed to the sufficient attraction between zinc ions and the hydroxyl groups (−OH) on PVA, facilitating the formation of well-defined rhombic dodecahedral crystals.^30,40^ Transmission electron microscopy (TEM) and scanning electron microscopy (SEM) images reveal that the characteristic rhombic dodecahedral morphology of ZIF-8 cannot form under room temperature conditions with a 6-h reaction time unless poly­(vinyl alcohol) (PVA) is introduced as a nucleating agent (Figure 2 **Ai**). In contrast, under identical conditions, the addition of PVA markedly promotes the formation of well-defined rhombic dodecahedral ZIF-8 crystals with an average size of 356.8 ± 44.3 nm (Figure 2 **Aii**). Furthermore, as shown in Figure 2 **Aiii**, substituting PVA with Aromatase as the core component similarly results in the formation of uniform rhombic dodecahedral crystals (Aromatase@ZIF-8) with a slightly larger average size of 476.6 ± 61.2 nm. Compared to PVA@ZIF-8, Aromatase@ZIF-8 crystals exhibit a smoother surface and greater size, likely due to the absence of PVA intercalation and the encapsulation of Aromatase within the ZIF-8 framework. Additionally, when CDX and Aromatase were cointroduced during ZIF-8 synthesis, the resulting ZIF-8 crystals exhibited a further increase in size to 650.1 ± 63.6 nm (Figure 2 **Aiv**), indicating successful coencapsulation of CDX and Aromatase within the ZIF-8 structure.

**Figure 2:**
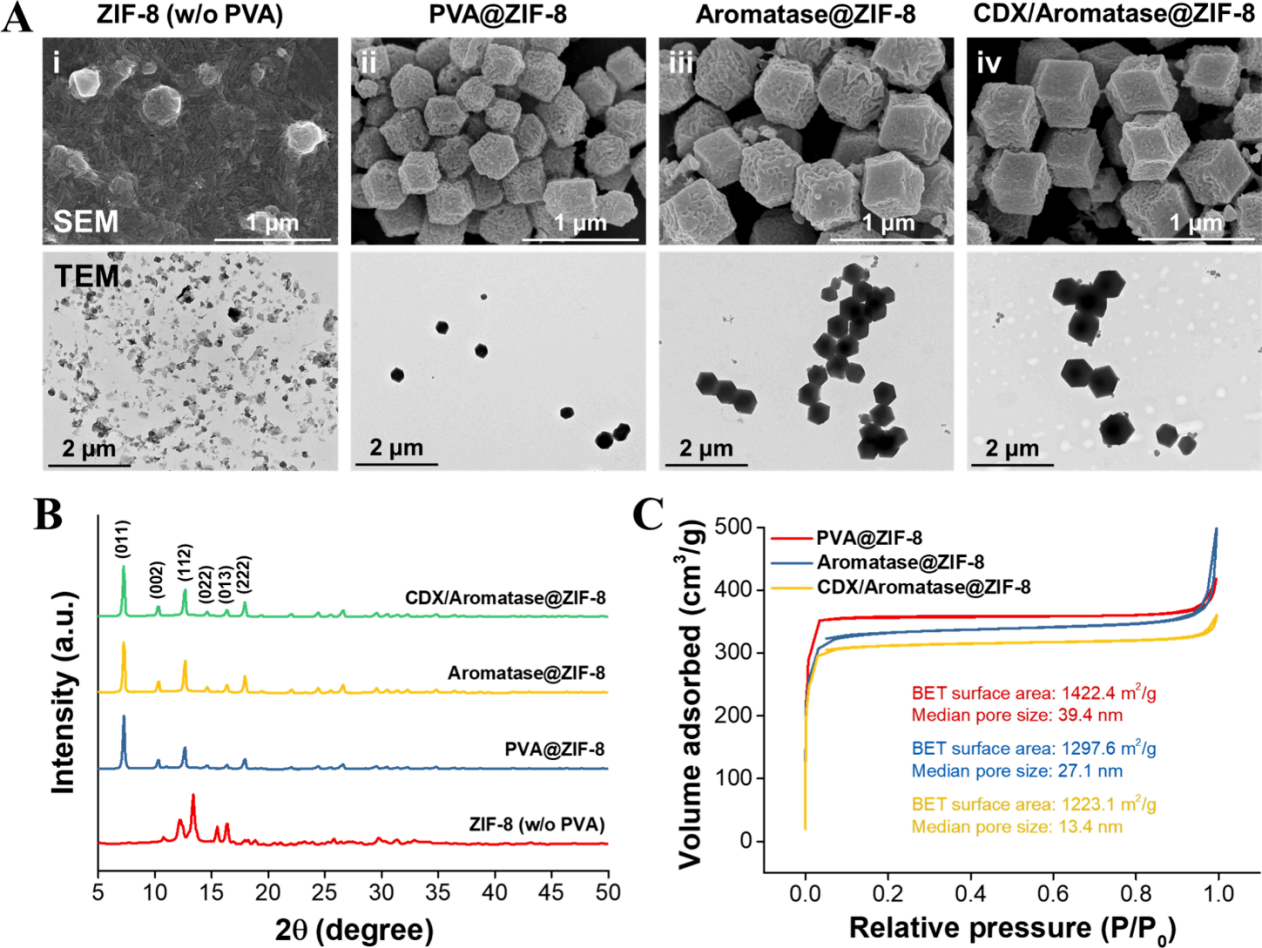
(A) SEM (top) and TEM (bottom) images of ZIF-8 (without PVA), PVA@ZIF-8, Aromatase@ZIF-8, and CDX/Aromatase@ZIF-8 synthesized through the bulk solution. (B) XRD pattern of ZIF-8 (without PVA), PVA@ZIF-8, Aromatase@ZIF-8, and CDX/Aromatase@ZIF-8. (C) Nitrogen adsorption–desorption isotherms for PVA@ZIF-8, Aromatase@ZIF-8, and CDX/Aromatase@ZIF-8 exhibit Type I behavior indicative of microporous structures.

The structural integrity of ZIF-8 (without PVA), PVA@ZIF-8, Aromatase@ZIF-8, and CDX/Aromatase@ZIF-8 was verified via X-ray diffraction (XRD) analysis (Figure 2B). Distinct peaks observed at Bragg angles (2θ) = 7.3°, 10.4°, 12.7°, 14.7°, 16.5°, and 18.0°, corresponding to the Miller indices of the (011), (002), (112), (022), (013), and (222) planes, respectively, confirm the high crystallinity of the synthesized ZIF-8 with a rhombic dodecahedral morphology. The XRD patterns of PVA@ZIF-8, Aromatase@ZIF-8, and CDX/Aromatase@ZIF-8 exhibit similar characteristic peaks to those of pristine ZIF-8, indicating that the crystal structure remains intact following the encapsulation of CDX and Aromatase. The Brunauer–Emmett–Teller (BET) analysis was conducted to evaluate changes in the specific surface area and pore size of ZIF-8 after the encapsulation of Aromatase or CDX/Aromatase (Figure 2C). The results revealed a reduction in pore size from 39.4 to 27.1 nm (a decrease of approximately 31.2%) and a decrease in specific surface area from 1422.4 m^2^/g to 1297.6 m^2^/g (approximately 8.8%) in the Aromatase@ZIF-8 group, confirming the successful encapsulation of Aromatase within ZIF-8. In the CDX/Aromatase@ZIF-8 group, the pore size further decreased to 13.4 nm (a reduction of approximately 65.9%), and the specific surface area decreased to 1223.1 m^2^/g (approximately 14.1%), indicating the effective coencapsulation of CDX and Aromatase within the ZIF-8 framework.

To ensure maximum drug delivery per RLMNP, it is necessary to investigate and identify the ZIF-8 preparation parameters that offer the highest drug loading efficiency. Therefore, we prepared CDX/Aromatase@ZIF-8 using varying concentrations of 2-MIM (3, 6, 12, and 24 mg/mL) while maintaining constant concentrations of Aromatase (0.25 mg/mL), CDX (1 mg/mL), and ZnCl_2_ (5 mg/mL). The results demonstrated that increasing the 2-MIM concentration from 3 mg/mL to 24 mg/mL significantly improved the yield of CDX/Aromatase@ZIF-8 (2.4, 2.9, 3.7, and 5.6 mg, respectively). However, the drug loading efficiency decreased progressively, with values of 20.6, 17.1, 13.4, and 8.8 wt %, respectively. The amounts of CDX loaded per milligram of CDX/Aromatase@ZIF-8 were 0.2, 0.17, 0.13, and 0.08 mg, while the corresponding Aromatase contents were 0.04, 0.03, 0.02, and 0.01 mg, respectively. Therefore, in this study, we selected a 2-MIM concentration of 3 mg/mL as the optimal condition for the preparation of CDX/Aromatase@ZIF-8. Under this condition, each milligram of CDX/Aromatase@ZIF-8 contains 0.2 mg of CDX and 0.04 mg of Aromatase.

In this study, CDX/Aromatase@ZIF-8 was incorporated into the short-term release layer of the RLMNP. Since the preparation of RLMNP requires suspending CDX/Aromatase@ZIF-8 in an aqueous solution, ensuring its stability and dispersibility in water is essential. To enhance these properties, 1 wt % PVA was added to the CDX/Aromatase@ZIF-8 solution. Experimental results demonstrated that sedimentation made the supernatant in the untreated CDX/Aromatase@ZIF-8 group (without PVA) progressively clearer over time. Noticeable precipitation occurred within 1 h, and by 6 h, the CDX/Aromatase@ZIF-8 had completely settled at the bottom of the cuvette. In contrast, the PVA-treated CDX/Aromatase@ZIF-8 solution remained uniformly dispersed, with no visible sedimentation observed even after 6 h (Figure 3A). These findings indicate that treating CDX/Aromatase@ZIF-8 with 1 wt % PVA significantly improves its suspension stability and dispersibility in aqueous media. The enhanced performance is likely attributed to the interaction between the numerous hydroxyl (−OH) groups of PVA and the surface of CDX/Aromatase@ZIF-8.

**Figure 3:**
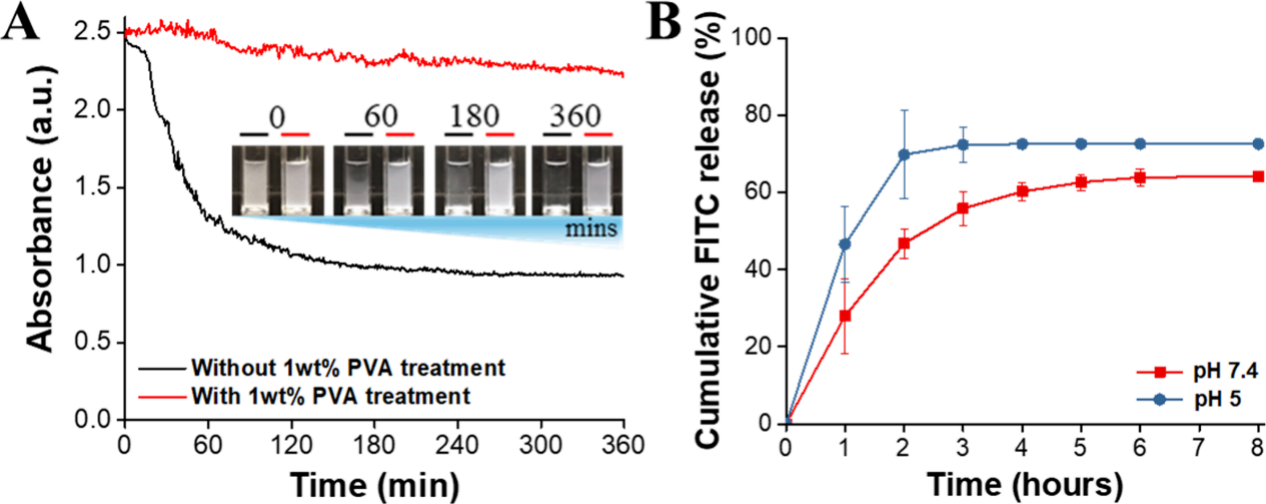
(A) UV/vis spectroscopy analysis of CDX/Aromatase@ZIF-8, with and without PVA, to evaluate aqueous dispersibility of CDX/Aromatase@ZIF-8. The inset presents dispersion imaging results captured at various time intervals. (B) Cumulative release profiles of FITC from FITC/Aromatase@ZIF-8 at pH 5.0 and pH 7.4 over an 8-h period.

To investigate the pH-responsive behavior of CDX/Aromatase@ZIF-8 and evaluate its in vitro release profile for rapid drug delivery, FITC was used as a substitute for CDX to form FITC/Aromatase@ZIF-8, enabling assessment of FITC release efficiency. The results indicated that under acidic conditions (pH 5), the cumulative release rates within the first 3 h were 46.5 ± 9.8%, 69.8 ± 11.3%, and 72.3 ± 4.6%, demonstrating that the majority of the drug was released during this period. In contrast, at physiological pH (7.4), the cumulative release rates within the first 3 h were 27.9 ± 9.6%, 46.7 ± 3.8%, and 55.8 ± 4.4%, with sustained release reaching 64.2 ± 0.3% by the eighth hour (Figure 3B). A comparative analysis revealed that the 2-MIM component in FITC/Aromatase@ZIF-8 undergoes protonation more readily in acidic environments, leading to structural disintegration. This phenomenon leads to an initial burst release and a greater cumulative release of CDX from CDX/Aromatase@ZIF-8 under the acidic conditions of the tumor microenvironment.

### 3-3 *In Vitro* Hormone Therapy Efficiency of CDX/Aromatase@ZIF-8

Upon degradation of ZIF-8 within cells in response to pH changes, the released zinc ions accumulate intracellularly. As the intracellular zinc concentration rises, it promotes an increase in reactive oxygen species (ROS) levels while inhibiting glutathione reductase (GR), an enzyme crucial for the regeneration of the antioxidant glutathione (GSH). The inhibition of GR leads to a reduction in GSH levels, further amplifying ROS accumulation. Elevated ROS levels subsequently induce the upregulation of pro-inflammatory cytokines, including CCL4 and IL6, which ultimately result in cell necrosis. In the initial phase of zinc accumulation, genes such as MT1A and CASP9 are upregulated to facilitate zinc detoxification.^32,41^ However, the rate of zinc accumulation surpasses the cellular expulsion capacity, culminating in cell necrosis. Therefore, it is essential to assess the cytotoxicity of ZIF-8 on two PCa cell lines, LNCaP and CWR22R. Results indicated that ZIF-8 demonstrated high biocompatibility at concentrations up to 75 μg/mL for both cell lines, with cell viability maintained above 97% (97.9 ± 0.9% for LNCaP; 97.3 ± 0.8% for CWR22R). At higher concentrations, specifically at 100 μg/mL and 125 μg/mL, a notable decrease in viability was observed, suggesting mild cytotoxicity at these elevated doses (Figures 4A, 4D).

**Figure 4:**
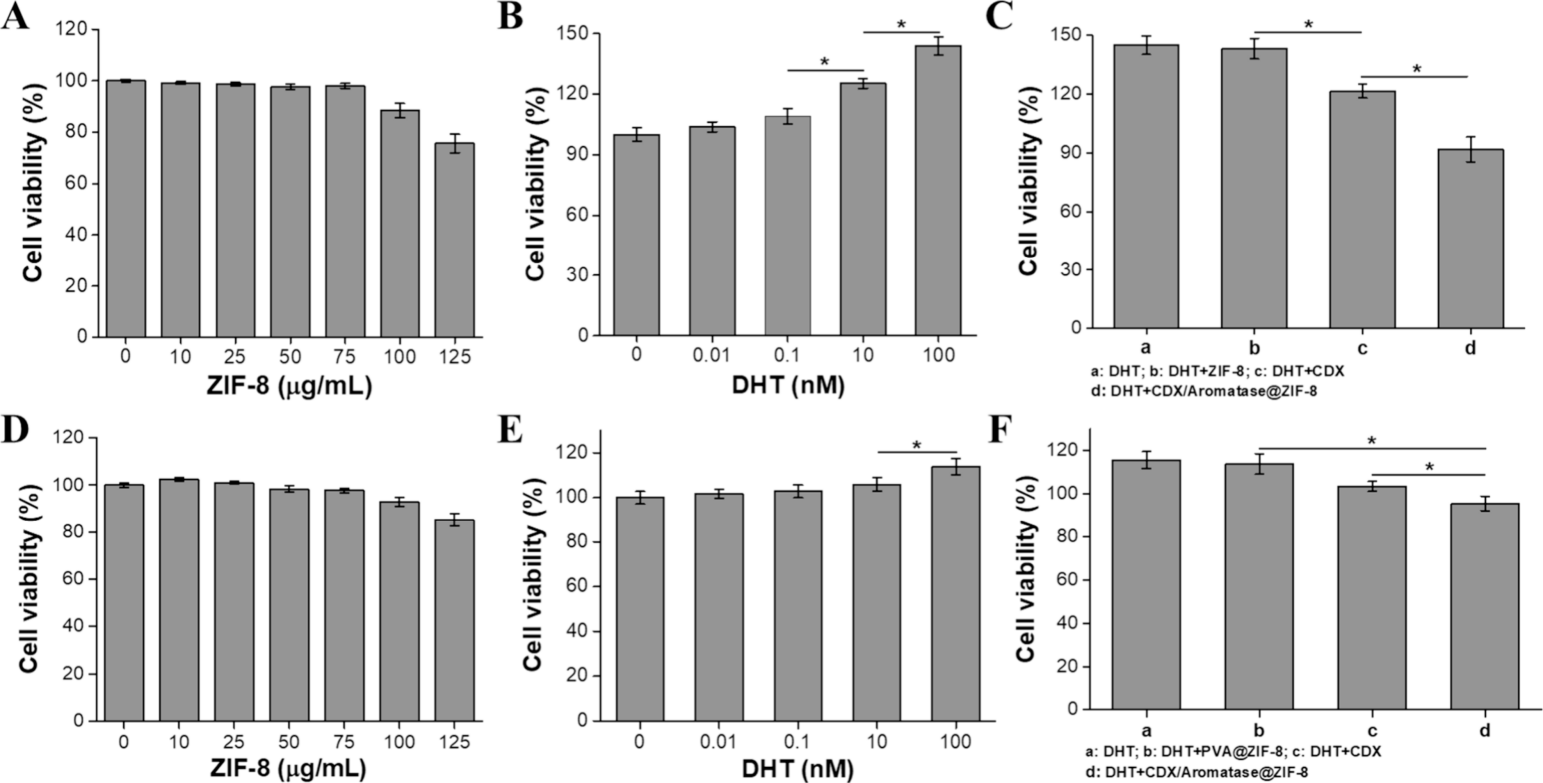
(A) Cell viability of LNCaP cells following 24-h coculture with varying concentrations of pure ZIF-8 containing PVA. (B) Cell viability of LNCaP cells after 48-h coculture with different concentrations of DHT. (C) Cell viability of LNCaP cells after 48 h of coculture under the following conditions: (a) Control group (100 nM DHT), (b) DHT + ZIF-8, (c) DHT + CDX (10 μM CDX), and (d) DHT + CDX/Aromatase@ZIF-8 (10 μM CDX and 50 μg/mL ZIF-8). (D) Cell viability of CWR22R cells following 24-h coculture with varying concentrations of pure ZIF-8 containing PVA. (E) Cell viability of CWR22R cells after 48-h coculture with different concentrations of DHT. (F) Cell viability of CWR22R cells after 48 h of coculture under the following conditions: (a) Control group (100 nM DHT), (b) DHT + ZIF-8, (c) DHT + CDX (10 μM CDX), and (d) DHT + CDX/Aromatase@ZIF-8 (10 μM CDX and 50 μg/mL ZIF-8). Data are expressed as mean ± SD (*N* = 6), with significant differences denoted by asterisks (Student’s *t* test, *p ≤ 0.05).

Testosterone and DHT are key androgens that have been shown to stimulate the proliferation of PCa cells and are commonly detected in patients with the disease.^42,43^ To mimic *in vivo* conditions, LNCaP and CWR22R cells were cultured with varying concentrations of DHT for 48 h to assess the dose-dependent effects of DHT on cell proliferation *in vitro*. Both cell lines exhibited increased cell viability upon treatment with escalating concentrations of DHT (0.01 to 100 nM). The highest concentration tested (100 nM) significantly stimulated cellular proliferation, with viability increasing by approximately 43.9 ± 4.5% and 13.7 ± 3.7% above baseline in LNCaP and CWR22R cells, respectively (Figures 4 **B,** [Fig fig4]E). Consequently, a DHT concentration of 100 nM was selected for subsequent experiments to model androgen-driven proliferation. CDX is currently utilized in clinical hormone therapy for PCa. Its primary mode of action involves penetrating target cells and competitively binding to androgen receptors, thereby inhibiting the binding of DHT. This competitive inhibition suppresses cellular proliferation and decreases overall cell viability.^44^ We further examined the inhibitory potential of CDX and CDX/Aromatase@ZIF-8 on androgen receptor-mediated proliferation in both PCa cell lines. Treatment with free CDX notably reduced the viability of DHT-stimulated LNCaP cells from 144.7 ± 4.7% to 121.3 ± 3.6%. Remarkably, CDX/Aromatase@ZIF-8 demonstrated superior inhibitory efficacy, further decreasing viability to 91.7 ± 6.3%, indicative of enhanced intracellular delivery and efficacy of CDX (Figure 4C). A similar trend was observed in CWR22R cells, although the overall impact was slightly less pronounced due to their lower androgen responsiveness (from 115.7 ± 3.9% to 95.6 ± 3.3%). Nevertheless, CDX/Aromatase@ZIF-8 treatment significantly reduced viability compared to free CDX treatment alone, demonstrating consistent effectiveness across different PCa cell models (Figure 4F). Collectively, these findings underscore ZIF-8’s excellent biocompatibility and its potential to enhance therapeutic efficacy when employed as a carrier for androgen receptor inhibitors like CDX. The results suggest significant promise for the development of CDX/Aromatase@ZIF-8 as effective therapeutic strategies for PCa treatment, even in PCa models with varying androgen sensitivity.

### 3-4 Characterization of RLMNP

For ease of administration, we further loaded the free Goserelin and CDX/Aromatase@ZIF-8 into a microneedle patch (MNP) to form RLMNP, which were arranged in a 10 × 10 array with an area of 10 × 10 mm and backed for stability during handling using medical tape for self-administered, painless, and sustained hormonal therapy. To investigate the distribution of free Goserelin and CDX/Aromatase@ZIF-8 within the RLMNP system, FITC was used as a model for free Goserelin, while RhB/Aromatase@ZIF-8 simulated CDX/Aromatase@ZIF-8. Each microneedle was conical in shape, with a base diameter of 500 μm, a height of 840 μm (including the needle length of 690 μm and a funnel-shaped base of 150 μm), and a tip radius of approximately 10 μm. A fluorescence microscope was employed to examine the layer-specific distribution of FITC (green fluorescence, representing free Goserelin) and RhB/Aromatase@ZIF-8 (red fluorescence, representing CDX/Aromatase@ZIF-8) within the RLMNP structure. In this study, we formulated rapidly dissolvable RLMNP using highly water-soluble PVA/PVP to enable efficient transdermal delivery of Goserelin/Alg-Tyr hydrogel and CDX/Aromatase@ZIF-8. Each RLMNP contains approximately 0.28 mg of Goserelin and 0.04 mg of CDX.

The fluorescence image in Figure 5 **A (left)** shows that FITC with Alg-Tyr hydrogel (FITC/Alg-Tyr hydrogel) was uniformly localized at the tips of the RLMNP. This observation was further corroborated by vertical SEM imaging, as depicted in Figure 5 **B**. Upon needle sectioning, the second layer, which contained RhB/Aromatase@ZIF-8, exhibited a rougher fracture surface and numerous spherical structures (Figure 5 **B, right**) compared to the smoother fracture surface of the first layer (containing FITC/Alg-Tyr hydrogel), as shown in Figure 5 **B (left)**. Furthermore, Figure 5 **A (middle)** illustrates that RhB/Aromatase@ZIF-8 was homogeneously distributed within the remaining needle volume, and the merged fluorescence image (Figure 5 **A, right**) distinctly highlights the two-layered microneedle structure. Measurement of the mechanical strength using a tensile compression machine indicated that the failure force for the RLMNP without cross-linking was 0.015 N per needle, which was significantly lower than the RLMNP with cross-linking (0.29 N per needle), indicating that the Alg-Tyr solution filled the needle tip region and requires cross-linking to form a stable Alg-Tyr hydrogel, thereby significantly enhancing its mechanical strength and improving its skin penetration efficiency (Figure 5C). In addition, the mechanical strength of the RLMNP is much larger than the force reportedly required to puncture human skin (∼0.058 N/needle),^45^ indicating that the fabricated RLMNP would be strong enough to penetrate the skin. After insertion of the RLMNP into porcine cadaver skin, all generated cavities filled with FITC/Alg-Tyr hydrogel and RhB/Aromatase@ZIF-8 that could not be wiped with ethanol from the skin’s surface (Figure 5D), indicating that the stiffness of the RLMNP was mechanically robust enough to withstand insertion into porcine cadaver skin and then release FITC/Alg-Tyr hydrogel and RhB/Aromatase@ZIF-8 into each generated cavity.

**Figure 5:**
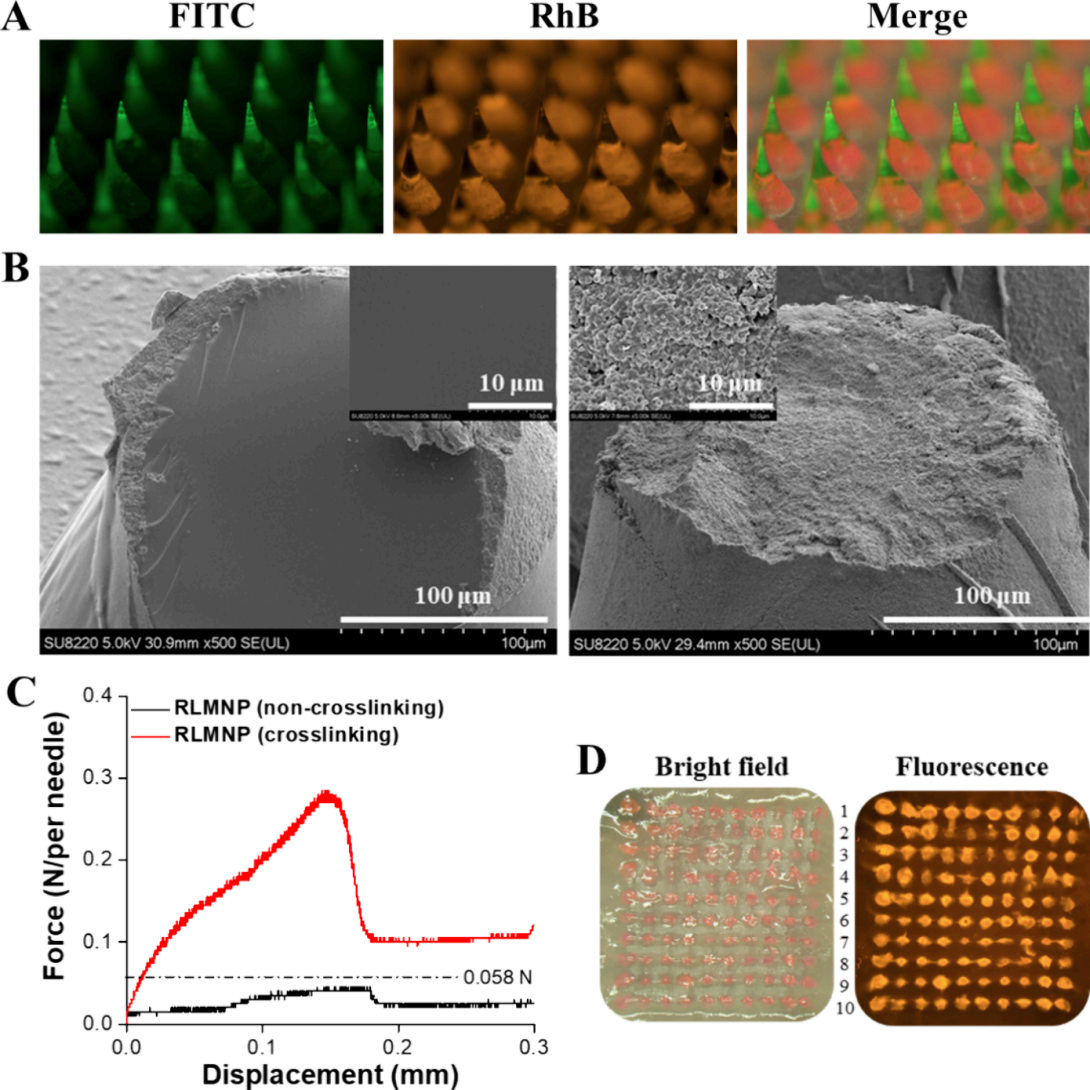
(A) Representative fluorescence microscopy images of RLMNPs with a PVA/PVP base. (B) Cross-sectional SEM images of RLMNPs: A side view of the Goserelin/Alg-Tyr hydrogel cross-section at the upper end with its magnified view (left), and a side view of the CDX/Aromatase@ZIF-8 cross-section near the base with its magnified view (right). (C) Mechanical behavior of non-cross-linked (black curve) and cross-linked (red curve) RLMNPs. (D) Bright-field and fluorescence microscopy images demonstrating the formation of microcavities and the successful delivery of FITC and RhB/Aromatase@ZIF-8 into porcine cadaver skin.

### 3-5 *In Vitro* Studies of RLMNP Penetration and Dissolution

To investigate the delamination behavior of microneedles following RLMNP penetration into the skin, RLMNP loaded with FITC/Alg-Tyr hydrogel (first layer; needle tip region) and RhB/Aromatase@ZIF-8 (second layer; residual region) was inserted into 1% agarose gel, and the distribution of FITC/Alg-Tyr hydrogel and RhB/Aromatase@ZIF-8 within the agarose gel was examined using fluorescence microscopy. After administration, the horizontal direction images of 1% agarose gel at the administration sites were recorded using fluorescence microscopy. The second layer of RLMNP rapidly dissolved upon administration, releasing RhB/Aromatase@ZIF-8 (red fluorescence), which subsequently diffused from the initial penetration site to the surrounding region. As a result, prominent red fluorescence was observed throughout the entire agarose gel after 30 min of administration. Simultaneously, the released FITC/Alg-Tyr hydrogel (green fluorescence) was observed to accumulate at the site of RLMNP penetration, retaining its triangular cone-shaped structure. Furthermore, FITC was gradually and continuously released from the Alg-Tyr hydrogel and diffused into the surrounding area (Figure 6A), revealing that RLMNP is capable of immediate CDX/Aromatase@ZIF-8 release to block androgen-DHT interaction and sustained release of Goserelin to down-regulate the androgen production for enhanced hormonal therapy.

**Figure 6:**
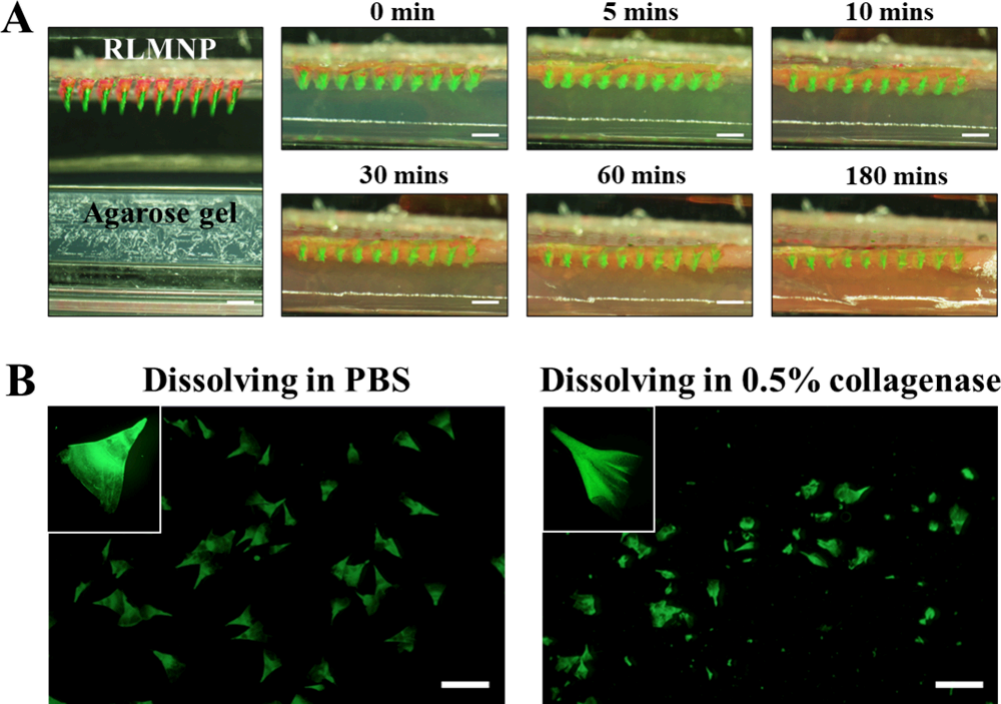
(A) Composite image analysis illustrating the in vitro simulated release and penetration of RLMNP into 1% agarose gel over time intervals of 5, 10, 30, 60, and 180 min (scale bar = 1 mm). (B) Dissolution behavior of RLMNP in PBS solution (left) compared to PBS solution containing 0.05% collagenase (right) over a 24-h period (scale bar = 1 mm).

To confirm that the sustained-release hydrogel layer (first layer) of RLMNP would not dissolve immediately after administration, the RLMNP was placed in a microtube containing PBS solution (pH 7.4) or PBS solution containing 0.05% collagenase (Figure 6B). The results indicated that only a triangular cone-shaped hydrogel layer was observed after adding RLMNP to the PBS solution, confirming that the Alg-Tyr hydrogel synthesized in this study could cross-link and aggregate at the tips of the microneedles in the PDMS mold. To simulate the subcutaneous tissue environment, 0.05% collagenase was added to the PBS solution (pH 7.4), and RLMNP was immersed in a microtube containing this solution for 24 h. After incubation, significant morphological changes and notable shrinkage of the triangular cone-shaped hydrogel layer were observed. These findings demonstrate that the Alg-Tyr hydrogel can undergo gradual enzymatic degradation, facilitating sustained Goserelin release. These findings demonstrate that the RLMNP functions as an efficient platform for codelivering two drugs with distinct mechanisms of action, enabling both rapid and sustained drug release, thereby improving the efficacy of hormone therapy for PCa.

### 3-6 *In Vivo* PCa Hormone Therapy Using RLMNP

To evaluate the efficacy of RLMNP-mediated transdermal delivery of Goserelin, serum androgen levels were measured at multiple time points following treatment. As shown in Figure 7A, baseline androgen levels on Day 0 were approximately 11.6 ± 1.3 nM. Following the first RLMNP application, androgen levels remained stable on Day 4 (10.8 ± 1.9 nM), but a significant flare-up was observed on Day 8, reaching a peak of approximately 18.4 ± 2.4 nM. Subsequently, androgen levels declined markedly: by Day 12, levels dropped to 7.7 ± 1.3 nM, followed by further reductions to 4.8 ± 1.8 nM and 2.1 ± 1.5 nM on Days 16 and 20, respectively. The significant decrease between Day 8 and Day 20 confirms that RLMNP can effectively deliver Goserelin through the skin, achieving sustained androgen suppression comparable to chemical castration. These results demonstrate the potential of RLMNP as a minimally invasive platform for long-acting hormonal therapy.

**Figure 7:**
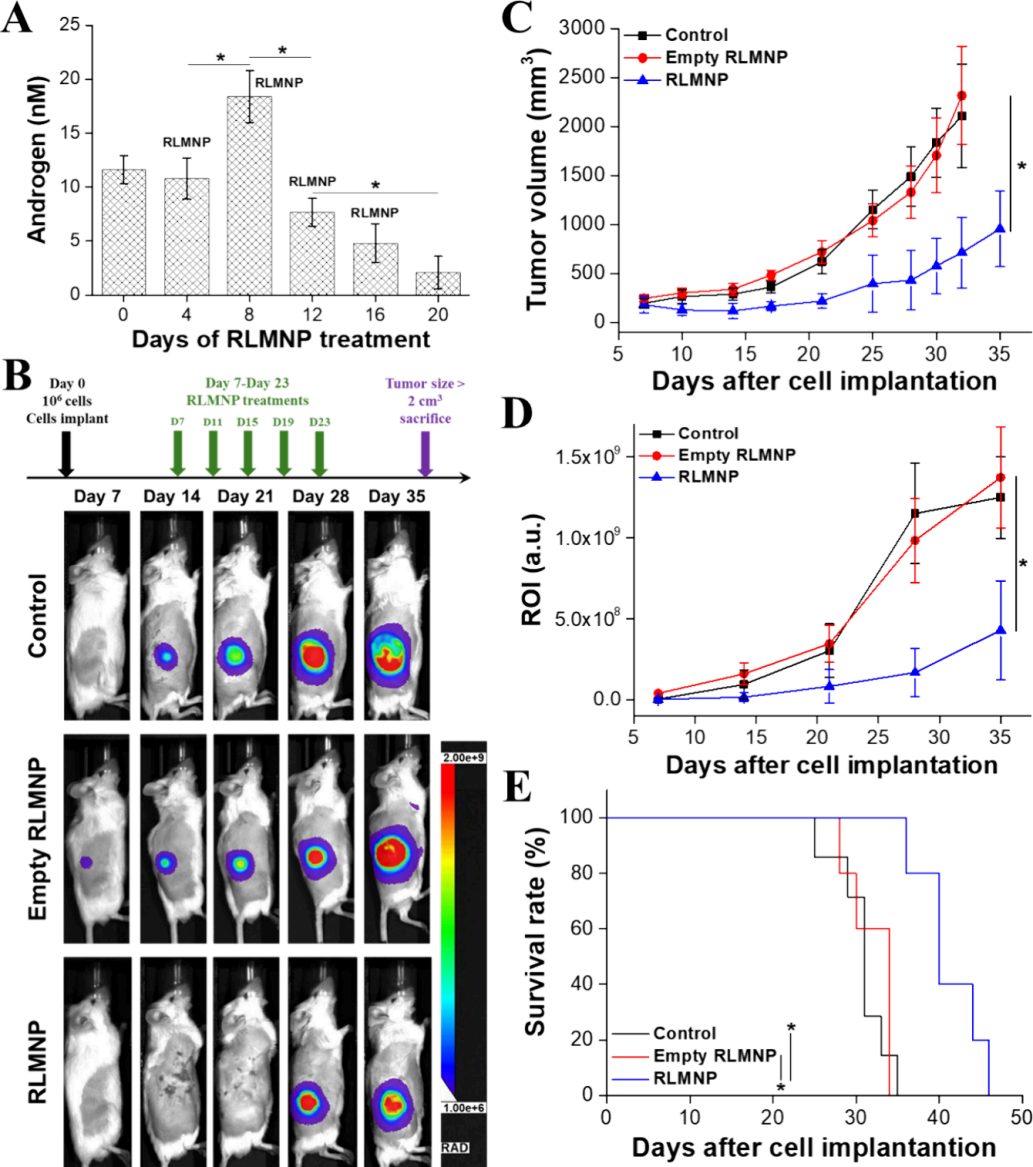
(A) Androgen levels in response to RLMNP treatment over time. Normal mice were treated with RLMNP, and androgen concentrations were measured at various time points (0, 4, 8, 12, 16, and 20 days). Data are represented as mean ± SD (*N* = 3), with significant differences denoted by asterisks (Student’s *t* test, *p ≤ 0.05). (B) Treatment protocols evaluating transdermal hormone therapy using RLMNP. Animals were monitored through IVIS imaging at the specified time points; representative images of one mouse from each group were shown. (C) Quantitative analysis of tumor size following various treatments. Data are presented as mean ± SD. Statistical significance was determined using Student’s *t* test (*p ≤ 0.05). (D) Tumor progression among treatment groups. Tumor volume ratios were calculated based on the luminescent intensity values obtained from IVIS imaging in (B) at each time point relative to day 7. Data are expressed as mean ± SD. Statistical significance was determined using Student’s *t* test (*p ≤ 0.05). (E) Survival curves of experimental animals. Transdermal hormone therapy using RLMNP significantly suppressed tumor progression and prolonged survival compared to the control groups. Data are represented as mean ± SD. Statistical significance was determined using Student’s *t* test (*p ≤ 0.05). Animals were euthanized when the implanted tumor volume reached 2 cm^3^. Tumor growth and survival rate were assessed in the same cohort of animals (control group: *N* = 7; empty RLMNP group: *N* = 5; RLMNP group: *N* = 5).

Treatment efficacy using RLMNP was evaluated in mice with hypodermic tumors induced by the injection of CWR22R cells. To assess the therapeutic effects of repeated RLMNP treatments, tumor-bearing mice were subjected to five treatments administered on Days 7, 11, 15, 19, and 23. Bioluminescence imaging (BLI) was employed to monitor tumor progression *in vivo*. The untreated control group and empty RLMNP (without Goserelin and CDX loading)-treated group exhibited a steady increase in BLI signal intensity, indicating rapid tumor growth. In contrast, the RLMNP (with Goserelin and CDX loading)-treated group showed slower progression of the BLI signal, demonstrating the therapeutic effect of the treatment in suppressing tumor growth (Figure 7B). Quantitative tumor volume analysis further validated the therapeutic effect of RLMNP. In both the control group and the group treated with empty RLMNPs, tumor volumes increased exponentially, surpassing the critical threshold of 2000 mm^3^ by day 30 post-cell implantation, and reaching 2105.1 ± 527.6 mm^3^ and 2315.2 ± 498.8 mm^3^, respectively, by day 32. In contrast, the tumor volume in the RLMNP treatment group exhibited significantly slower growth, reaching only 954.9 ± 387.3 mm^3^ after 35 days of cell implantation. This finding confirms that RLMNP effectively inhibits tumor growth through transdermal delivery of Goserelin and CDX, demonstrating its potential as a hormone therapy strategy (Figure 7C).

The luminescent intensity results corroborated the BLI and tumor volume findings. The untreated control group (Figure S1) and the group treated with empty RLMNPs (Figure S2) showed a consistent increase in luminescent intensity, indicating rapid tumor metabolic activity. Conversely, the RLMNP-treated group demonstrated a significantly slower increase in luminescent intensity, suggesting controlled tumor progression and potentially transdermal drug delivery effects mediated by the RLMNP system (Figure 7D). The median survival was significantly extended in animals receiving five doses of RLMNP treatment (40 days), compared with shorter median survival in the control group (31 days) and the empty RLMNP-treated group (34 days) (Figure 7E). Although the survival end point (tumor volume >2 cm^3^) was reached in both the control and empty RLMNP-treated groups by day 34, IVIS imaging was performed on a fixed weekly schedule. As the tumor volumes in these groups had not yet exceeded 2 cm^3^ by day 28, the mice remained alive on day 35, allowing luminescence data to be collected prior to euthanasia. Of note, in both the control group and the empty RLMNP-treated group, one mouse died unexpectedly before the tumor volume reached 2 cm^3^. For survival analysis, these spontaneously deceased mice were also included in the statistics to accurately reflect all animals monitored. Moreover, the RLMNP transdermal drug delivery system did not adversely affect the general health or appetite of treated mice. The observed changes in tumor size highlight the localized and sustained drug release capabilities of the RLMNP system, effectively mitigating tumor progression. The gradual degradation of the hydrogel facilitated the continuous release of Goserelin and CDX, ensuring therapeutic efficacy throughout the treatment period. The long-acting layer, comprising a sustained-release hydrogel loaded with Goserelin, inhibits testosterone production via hypothalamic-pituitary–gonadal axis modulation, depriving PCa cells of essential growth factors and thereby inhibiting their proliferation. Meanwhile, the short-acting layer, constructed from pH-responsive ZIF encapsulating the hydrophobic drug CDX, directly targets PCa cells by competing with DHT for androgen receptor binding. Upon binding, CDX suppresses the translation of proteins essential for PCa cell growth, further delaying tumor progression. By integrating microneedle-based transdermal delivery with hydrogel-mediated sustained drug release, the RLMNP system offers significant advantages, including targeted therapeutic action, prolonged drug efficacy, and minimized systemic side effects.

Although this study primarily aimed to evaluate the overall therapeutic efficacy of the integrated dual-layer RLMNP system and has preliminarily demonstrated the distinct phase-release kinetics of each layer *in vitro*, the independent *in vivo* contribution of the short-acting (CDX-loaded ZIF-8) and long-acting (Goserelin-loaded hydrogel) layers was not separately quantified. This represents a limitation of the current study, as it prevents a precise understanding of how each release profile individually contributes to the overall therapeutic outcome. Future studies should include separate formulations or selective inhibition approaches to isolate and verify the individual *in vivo* effects of the short-acting and long-acting components, thereby enabling a more detailed mechanistic interpretation of their synergistic or complementary roles.

## 4 Conclusion

This study presents the development of an innovative rocket-like microneedle patch (RLMNP) for sustained hormone therapy in PCa, providing a dual-layer drug delivery platform that effectively integrates rapid-release and sustained-release mechanisms. The rapid-release layer, employing pH-responsive ZIF-8, delivers CDX to disrupt androgen receptor signaling and induce apoptosis in PCa cells. Meanwhile, the sustained-release layer, composed of Alg-Tyr hydrogel loaded with Goserelin, suppresses androgen synthesis by targeting the hypothalamic-pituitary–gonadal axis, thereby addressing both upstream and downstream pathways of hormone-driven tumor progression. The RLMNP demonstrates excellent mechanical strength, skin penetration capability, and biocompatibility. *In vivo* experiments reveal its significant therapeutic efficacy in reducing tumor volume, prolonging survival, and minimizing adverse effects. This painless, user-friendly, and self-administered system overcomes the challenges associated with traditional chemical or surgical castration, including patient discomfort, low compliance, and medical waste. In conclusion, the RLMNP represents a groundbreaking advancement in PCa management, offering a synergistic and targeted therapeutic solution with high clinical potential. By integrating state-of-the-art microneedle technology and tailored drug delivery strategies, this platform sets a new benchmark for treating hormone-sensitive malignancies.

## Supplementary Material



## Notes

Figure S1. *In vivo* bioluminescence IVIS imaging of tumor progression in the control group. Figure S2. *In vivo* bioluminescence IVIS imaging of tumor progression in mice treated with empty RLMNPs (PDF)
