# Supplementary material for: Rocket-Like Microneedle Patch for Sustained Hormone Therapy in Prostate Cancer
Source: ACS Biomater Sci Eng. 2025 Jul 18;11(8):4830–43. doi: 10.1021/acsbiomaterials.5c00127 (PMC12344640; doi:10.1021/acsbiomaterials.5c00127)
Supplement: Supplementary file 1 [file ab5c00127_si_001.pdf]

Supporting Information

Rocket-Like Microneedle Patch for Sustained Hormone Therapy in Prostate Cancer

Ying-Tzu Chen[1,2], Kuan-Ta Chen[3], Kai-Jie Yu[4,5], See-Tong Pang[4,5,6,*], Hung-Wei Yang[2,7,*]

[1]Department of Neurosurgery, Chang Gung Memorial Hospital, Linkou Branch, Taoyuan 33305, Taiwan

[2]Department of Biomedical Engineering, National Cheng Kung University, Tainan 70101, Taiwan

[3]Institute of Medical Science and Technology, National Sun Yat-sen University, Kaohsiung 80424, Taiwan

[4]Division of Urology, Department of Surgery, Chang Gung Memorial Hospital, Linkou Branch, Taoyuan 33305, Taiwan

[5]School of Medicine, College of Medicine, Chang Gung University, Taoyuan 33302, Taiwan

[6]Graduate Institute of Clinical Medical Science, College of Medicine, Chang Gung University, Taoyuan 33302, Taiwan

[7]Medical Device Innovation Center, National Cheng Kung University, Tainan 70101, Taiwan

Correspondence and requests for materials should be addressed to S.T. Pang (pst64lab@gmail.com) and H.W. Yang (howardyang@gs.ncku.edu.tw)

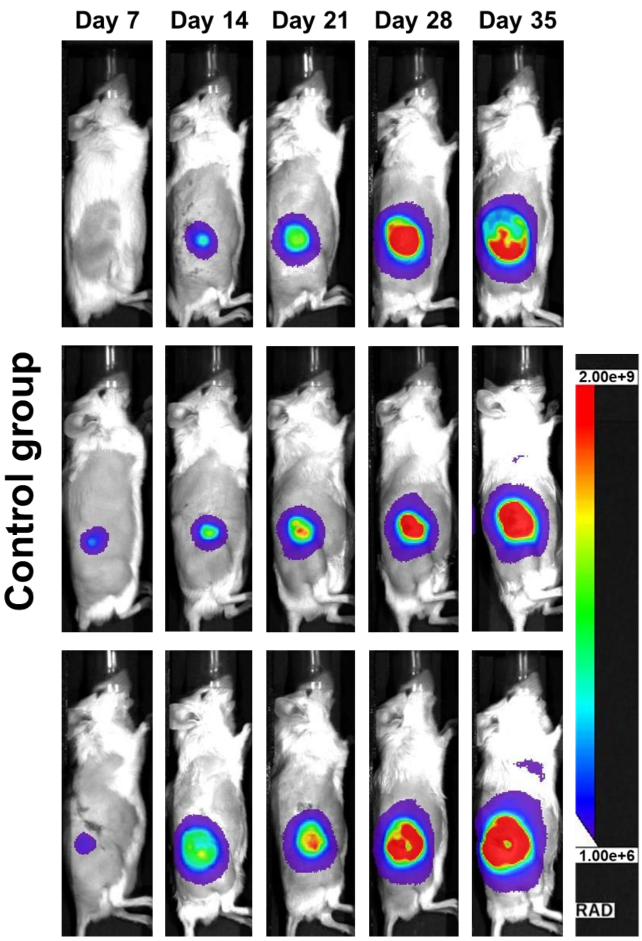

Supplementary Figure S1. *In vivo* bioluminescence IVIS imaging of tumor progression in the control group. The top row of images is identical to those shown in Figure 7B of the main text. Bioluminescence signals increased over time, indicating progressive tumor growth in the absence of treatment.

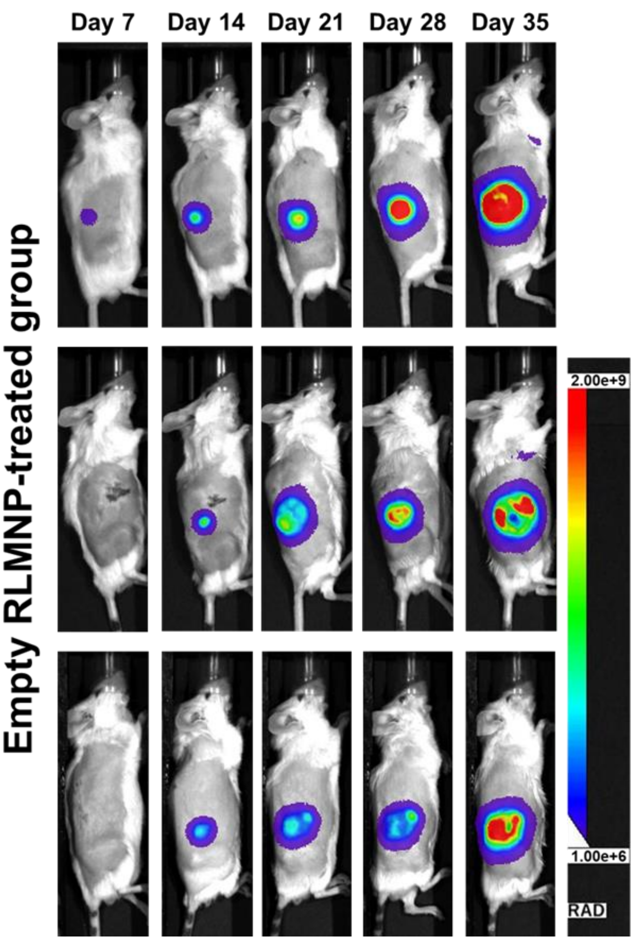

Supplementary Figure S2. *In vivo* bioluminescence IVIS imaging of tumor progression in mice treated with empty RLMNPs. The top row of images is identical to those shown in Figure 7B of the main text. Bioluminescence signals increased over time, indicating progressive tumor growth in the absence of therapeutic agents.
